# Multiple Routes to Oestrogen Antagonism

**DOI:** 10.3390/ph3113417

**Published:** 2010-10-29

**Authors:** Hilary R. Glover, Stewart Barker, Sylvanie D. M. Malouitre, John R. Puddefoot, Gavin P. Vinson

**Affiliations:** School of Biological and Chemical Sciences, Queen Mary, University of London, E1 4NS, UK

**Keywords:** oestrogen receptor, tamoxifen, trilostane, oestradiol, oestrone, oestriol, diethylstilboestrol, amplified in breast cancer, repressor of oestrogenic activity, MCF-7: T47D: LNCaP: PNT1A: ERE, hormesis, allosteric

## Abstract

Several lines of evidence attest to the existence of alternative ligand binding sites on the oestrogen receptor (ER), including non-competitive inhibition by trilostane or tamoxifen. It is possible that the inhibitory action of conventional oestrogen agonists at high concentrations may indicate that they too interact at alternative ER sites, albeit at low affinity. To test this possibility an oestrogen reporter assay was used to compare the activity of different oestrogens and antagonists in breast cancer and prostate cell lines. All four cell lines tested contained different amounts of oestrogen receptor α (ERα), ERβ, progesterone receptor and coregulator mRNA. Though differences were observed in response to stimulation and inhibition, these correlated only with the presence or absence of ERα, and not with the other components. Thus stimulation of the reporter by oestradiol and oestrone was biphasic in the breast cancer cells, while prostate cells were unable to respond. Only T47D cells were stimulated by oestriol or diethylstilboestrol, however reporter activity of all the cell lines was repressed by 10μM diethylstilboestrol. Reporter activity of MCF-7 cells was inhibited by tamoxifen, raloxifene and ICI 182,780, but stimulated by trilostane, yet all these antioestrogens inhibited agonist-stimulated activity. Trilostane also inhibited the agonism seen in cells co-treated with E2 and tamoxifen. It is clear that several of the compounds tested may have either agonist or antagonist effects under different conditions and at different concentrations, acting through ERα alone. Though biphasic dose response curves, or hormesis, have been attributed to various mechanisms, we here provide evidence that alternative ligand binding sites may contribute to this phenomenon.

## 1. Introduction

The steroid hormone oestradiol (E2) and the nuclear oestrogen receptors (ERα and ERβ) are involved in the regulation of many biological processes [1]. Critically, the interaction of oestrogen with its receptors is also involved in disease, especially breast cancer, and it is for this reason that oestrogen antagonists and blockers of oestrogen synthesis have been sought, and used therapeutically with great success [2,3].

Oestrogen acts by inducing a ligand specific alteration of ER conformation [4,5,6], thus increasing the stability of ER binding to an oestrogen response element (ERE) in the promoter of target genes and altering the recruitment of coregulatory proteins. This in turn permits reorganisation of DNA, leading to the recruitment of preinitiation complex [7]. ER activity is therefore tightly controlled by coregulators, coactivators and corepressors, which have ERα/β specificity and are regulated both by tissue distribution and by target gene promoter sequence (for reviews see [2,7,8,9]). Adding to the complexity of the oestrogenic response is the differential expression [10] and promoter specific activation [11] of ERα and ERβ. In addition, transactivation from other DNA elements, such as activator protein-1 (AP-1) [12] and sequence-specific DNA-binding protein-1 (Sp-1) [13] can be modified by ligand-bound ER, and ER has a further role by regulating cytoplasmic pathways such as MAPK and NO signalling [14].

In breast cancer, oestradiol has long been implicated in tumour proliferation and, since the 19th century, inhibition of E2 production has been a major therapeutic target [3,15]. Other treatments have focused on repression of ER activity using selective oestrogen receptor modulators (SERMS), which in some tissues retain oestrogenic activity, yet are antioestrogenic in the breast. Conversely, oestrogens, such as diethylstilboestrol (DES), are also effective as a treatment for breast cancer [16].

The antagonists that have so far been developed, including tamoxifen, raloxifene and ICI 182,780 [17,18,19,20,21] all interfere not only with gene transcription directly, but also they affect the recruitment of activators and repressors [22]. Since, however, they are all known to displace labelled oestradiol from the receptor, it is clear that they all bind to the same, classical, ligand binding site. Consequently, the fact that their actions are indeed selective, with differential effects on different tissues, presumably reflects their different efficacies in recruitment of other factors, such as coactivators/repressors, or the response may be opposed by other cell components, such as ERβ, present in variable amounts in different tissues. 

As more becomes known about ER function, other possibilities present themselves. Trilostane (Modrenal) has also been used in the treatment of breast cancer [23,24,25] and is effective in crossover studies with the aromatase inhibitor aminoglutethimide [26]. A striking characteristic of trilostane is that it is a non-competitive inhibitor of ER function [27], leading to the possibility that ligands interacting with allosteric sites on ER may contribute to the overall complexity of the oestrogenic response, and independently modulate coactivator/repressor involvement. 

Several other lines of evidence are consistent with the existence of second ligand binding domains in nuclear, particularly oestrogen receptors. Thus several polynuclear aromatic hydrocarbons were found to inhibit oestradiol stimulation of a yeast ER-reporter system, without competing for the primary ligand binding domain [28]. Jensen and Khan [29] proposed a two site model for oestrogen action, in which some non-steroidal antioestrogens such as tamoxifen bind to both the agonist ligand binding domain, and to a second, antagonist site, whereas pure antioestrogens, such as the steroid derivative fulvestrant bind only to the agonist site. This was held to explain the partial agonist actions of certain antioestrogens. Crystal structural evidence has been found for a second tamoxifen binding site in the coactivator binding site of the ERβ ligand binding domain [30,31]. 

The situation is complex, however. The polynuclear hydrocarbon tetrahydrochrysene (THC) and derivatives stimulated gene transcription by ERα in endometrial cancer cells, but depending on size and position of substituents, were antagonistic to ERβ [32,33], however, THC is known to bind to the ligand binding domain [34,35,36]. What then emerges here reflects the differences in ligand specificity and responses of ERα and ERβ [33] independent of any possible second site. It is here that consideration of trilostane’s action is again relevant. Not only is the inhibition of oestradiol stimulated response non -competitive, it too selectively stabilises ERβ [37], and moreover, it specifically induces ERβ gene transcription in MCF7 cells, as well as ERβ mRNA and protein expression in rat uteri [38], strongly illustrating the potential utility of this mode of therapy.

Possible indirect evidence for alternative ligand binding sites, may lie in the biphasic responses to oestrogen activation seen in mammalian cell lines [39]. Like similar examples of nonmonotonic responses in other systems, these have not been widely studied. However, models have been suggested that explain biphasic dose-response curves or hormesis, by variations in cell environment, gene damage or receptor homo and heterodimerisation [40,41] These studies did not consider the possibility that multiple ligand-receptor interactions might produce similar effects. 

To test the plausibility of these modes of action, the activities of various oestrogens were examined in ER positive breast and prostate cell lines, and the actions of trilostane and other SERMs were compared using an ERE-regulated reporter system. In this way subtle differences in the interactions between oestrogens and antioestrogens could be determined and compared to cellular levels of ERα, ERβ and PR and to variations in coregulator proteins. 

## 2. Experimental

### 2.1. Chemicals and general molecular methods

All the ER ligands and cell culture media used were purchased from Sigma-Aldrich Company Limited, Poole, UK apart from Raloxifene (Evista) which was from Eli Lilly and Co. Indianapolis, USA; Trilostane (Modrenal) from Stegram Pharmaceuticals Ltd, Billingshurst, UK; ICI 182,780 (Faslodex/ Fulvestrant) from Tocris, Ballwin, MO, USA.

### 2.2. Cell culture, transfection and luciferase assay

Two human breast cancer cell lines, MCF-7 and T47D, and two prostate lines, LNCaP and PNT1A (ECACC, Porton Down, UK) were routinely maintained in minimal essential medium eagle with Earl’s salts supplemented with 2mM L-glutamine, 1% non-essential amino acids (NEAA), 2% penicillin and streptomycin (P/S), 1mM sodium pyruvate and either 10% (MCF-7, LNCaP) or 5% (PNT1A, T47D) 5% foetal bovine serum. Prior to transfection just-confluent cells were seeded 1 in 2, into 96-well plates, so at 1.6 × 10^4^ cells per well. 

In order to remove compounding oestrogenic effects, 24 hours prior to transfection, media were replaced with phenol red-free Dulbecco’s minimal essential medium supplemented with 2mM L-glutamine, 1% NEAA, 2% P/S, 1mM sodium pyruvate and 10% charcoal stripped serum (Sera Laboratories International, Horsted Keynes, UK).

Construction of the reporter plasmid 3 × luc (three tandem consensus EREs) has been previously described [27]. EREs are usually found as half sites or imperfect palindromes which require extensive modelling of DNA for initiation of transactivation. The 3xERE system has been used because it gives a strong, measureable, response and reduces the role of co-regulator variation between cell lines. Transient transfections were performed in quadruplicate with 0.05μg reporter plasmid and 0.005μg of PRL-TK (Promega Corporation, Madison, WI, USA), which constitutively expresses *Renilla* luciferase and was included to correct for transfection variability, per well using Effectene (Qiagen Ltd., Crawley, UK). Concurrently with transfection, ligands were added to the cell growth medium and 10nM oestradiol and vehicle controls included. After 20 hours the cells were lysed, with passive lysis buffer, and frozen at -70°C. 

Luciferase activity was read using a Luminoskan Ascent (ThermoLabSystems, Altringham, UK). Two readings of 10 seconds were taken. The first gave firefly luciferase activity from the reporter and, after the addition of Stop and Glo reagent, the second reading gave *Renilla* luciferase activity from PRL-TK. Results were compared across transfections (RLU luciferin/RLU *Renilla*) as percentage of vehicle or 10nM oestradiol control. Results shown are the mean (±SEM) of at least 3 experiments of 3 or 4 replicates each. Statistical treatment of data was performed to produce average and standard error of mean for graphical representation. Student t-tests, one-way ANOVA and applicable post-tests, were performed using GraphPad Prism4 (GraphPad Software, Inc.San Diego, CA, USA).

### 2.3. RNA isolation and real-time RT-PCR (qRT-PCR)

Total RNA was isolated from cell lines using the RNA isolation kit, QIAamp (Qiagen Ltd.). 250ng total RNA from each cell line was analysed by real-time PCR using RT (superscript) III platinum one step RT-PCR enzymes (Invitrogen Life-Technologies Ltd., Paisley, UK) and TaqMan MGB, FAM labelled, probes (Applied Biosystems, Foster City, CA, USA) in a Mx3000P Real-Time PCR System instrument (Stratagene, La Jolla, CA, USA) and compared to a concentration curve from MCF-7 cells. The RNA was reverse transcribed at 50°C for 30 minutes Each PCR cycle consisted of denaturation at 95°C for 15 seconds and probe annealing at 60° for 30 seconds, after which fluorescence was measured and this was repeated for 40 cycles. Expression levels of endogenous control, large ribosomal protein (RPLPO), also determined by this method, were consistent across all the lines (data not shown

**Figure 1:**
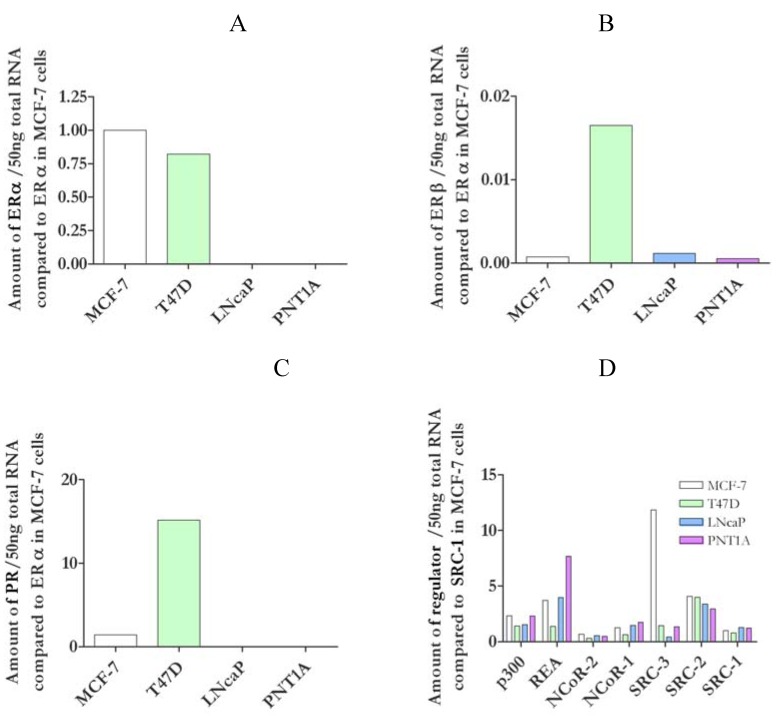
Expression levels of steroid receptor and co-regulator differ between cells lines. Amount of receptor mRNA present in 50ng of total RNA from each cell line measured by qRT-PCR. All values for ERα (A), ERβ (B) and PR (C) were expressed as ratios of ERα from MCF-7 cells. Amounts of coregulator mRNA (p300, REA, NCoR-2, SRC-3, SRC-2 and SRC-1) were expressed as a ratio of SRC-1 in MCF-7 cells (D). Each experiment was performed in duplicate and repeated three times.

## 3. Results and Discussion

### 3.1. Characterisation of nuclear receptors and coregulators in cell lines

Both of the breast cancer cell lines, MCF-7 and T47D, contained both ERα and ERβ mRNA (Figure 1a and Figure 1b). They had similar amounts of ERα and while ERβ levels were lower than ERα in both, T47D cells contained considerably more ERβ than MCF-7 cells. Both breast cancer derived cell lines were progesterone receptor (PR) positive (Figure 1c), with T47D containing much more PR than MCF-7 cells. In contrast the prostate cells (LNCaP and PNT1A) did not contain any ERα or PR and only had low levels of ERβ.

Levels of SRC-1, SRC-2/GRIP-1/TIF2, NCoR-1, NCoR-2/SMRT and p300 mRNA were comparable in the cell lines tested (Figure 1d). However MCF-7 contained eight times more AIB1/SRC-3 than T47D and PNT1A cells, and 28x more than LNCaP cells, and, while REA message was comparable between MCF-7 and PNT1A cells, it was 2.6 fold lower in T47D and 2 fold higher in PNT1A cells.

### 3.2. Oestrogen stimulated transcription was dependent upon cell type

**Figure 2:**
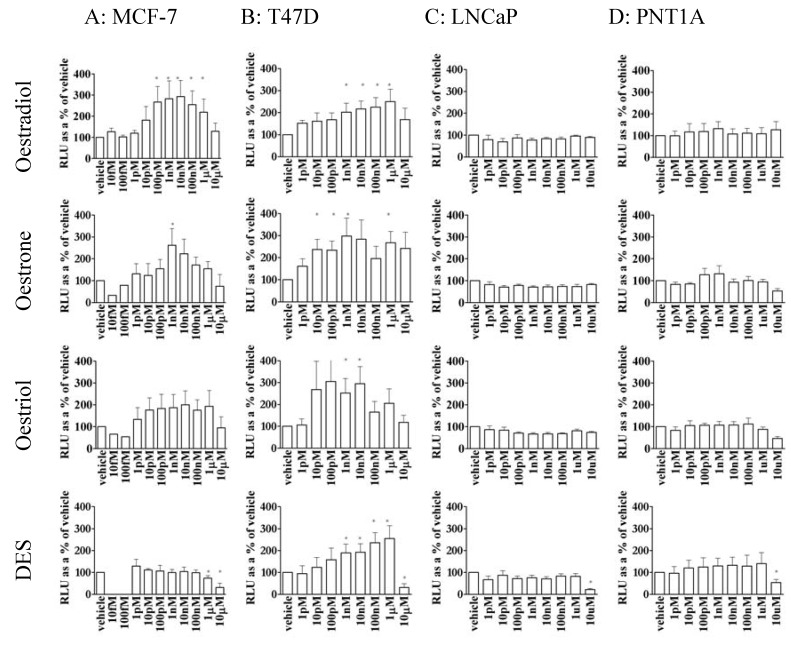
Oestrogen-stimulation of reporter activity in ER positive cell lines compared to basal activity in each cell line: MCF-7 cells (2A), T47D cells (2B), LNCaP cells (2C), and PNT1A cells (2D) stimulated with E2 (row 1), E1 (row 2), E3 (row 3) and DES (row 4). All the oestrogens tested slightly reduced LNCaP activity but significance is only shown for 10μM DES. * = significant (P<0.05) stimulation above control.

E2 stimulated reporter gene transactivation was determined in both breast cancer cell lines. E2 activity was increased by concentrations of 100 pM E2 and above in MCF-7 cells (Figure 2a). However reporter activity was biphasic and fell at concentrations of 100 nM E2 and higher, returning to basal levels at 10 μM. A similar pattern of activity was seen with T47D cells (Figure 2b) though these cells were not as sensitive to E2, as MCF-7 cells, requiring 1 nM E2 for stimulation. Again stimulation of the reporter was biphasic and the highest concentration of E2 used (10 μM) was not stimulatory. Reporter activity was not significantly stimulated by any concentration of E2 in either prostate cell line (Figure 2c and Figure 2d). None of the alterations in reporter activation could be attributed to differences in cell number or cells death as there was no significant alteration in the level of *Renilla* expression by treatment with ligands (data not shown). 

### 3.3. Oestrone, oestriol and DES were able to stimulate gene transcription in MCF-7 and T47D cells but not prostate cell lines

Since the reported receptor binding affinity for the other oestrogens such as oestrone (E1), oestriol (E3) and DES is not enough to describe the difference between their *in vitro* effects [42] these oestrogens were tested for their ability to transactivate reporter activity in this system. E1 was able to transactivate the reporter gene in MCF-7 and T47D cells (Figure 2a and Figure 2b). In MCF-7 cells E1 was not as active as E2, requiring 10x more ligand for stimulation but, similarly to E2, high concentrations of E1 dose-dependently reduced reporter activity. In contrast, T47D cells were more sensitive to E1 than E2 and, while neither E3 nor DES significantly stimulated MCF-7 cells both were active in T47D cells. In T47D cells E3 had the same dose-dependent pattern of reporter activity as E1, although the reduction in activity at high concentrations was more distinct, and DES had the same pattern as E2.

In contrast, neither LNCaP (Figure 2c) nor PNT1A (Figure 2d), was significantly stimulated by E2, E1, E3 or DES. In fact the highest concentration of DES tested (10 μM) repressed basal reporter activity in both of these cell lines as well as the breast cancer lines.

### 3.4. Except for trilostane, antioestrogens did not stimulate gene transcription in the absence of E2

**Figure 3:**
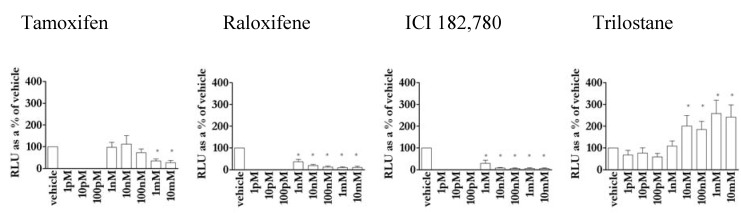
Antioestrogen (3A: tamoxifen, 3B: raloxifene, 3C: ICI 182,780 and 3D: trilostane) repression of basal reporter activity compared to basal activity in MCF-7 cells. * = significant stimulation/repression (P < 0.05) above/below basal.

None of the competitive SERMS, tamoxifen, raloxifene or ICI 182,780, stimulated reporter transactivation in MCF-7 cells in the absence of E2 (Figure 3). In fact they all suppressed basal activity. In contrast, 10 nM-10 μM of the non-competitive ligand trilostane was increasingly agonistic in this cell line. 

### 3.5. The action of antioestrogens was dependent on agonist in MCF-7 cells

**Figure 4:**
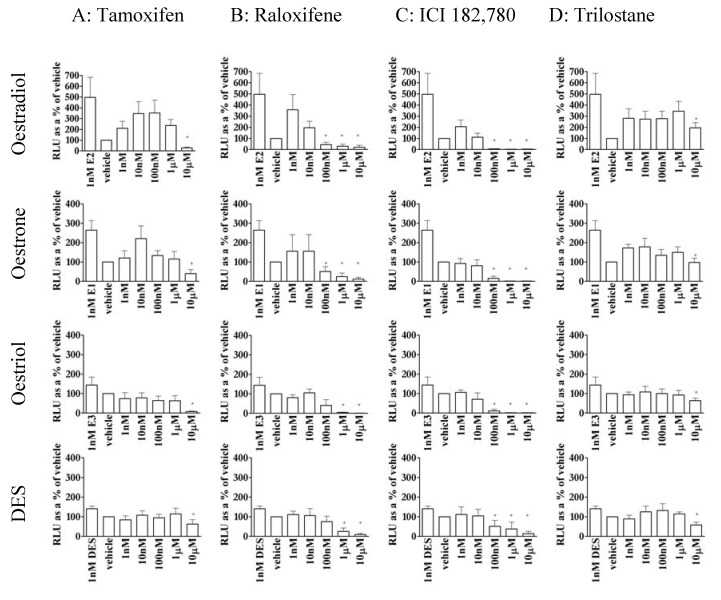
Antioestrogen repression of 1nM E2, E1, E3 or DES-stimulated reporter activity. Tamoxifen (4A), raloxifene (4B), ICI 182,780 (4C) and trilostane (4D) were co-treated with E2 (row 1), E1 (row2), E3 (row3) or DES (row4). Results are expressed as Relative Light Units (RLU) compared to vehicle-only wells (=100%). * = significant stimulation/repression (P < 0.05) above/below basal.

Tamoxifen (Figure 4a) was able to repress reporter activity in the presence of stimulatory levels (1 nM) of both E2 and E1 (though only at 10 μM) and, while 1 nM E3 and DES were inactive in this cell line, repression of reporter activity by tamoxifen in the presence of E3 or DES required ten fold more tamoxifen than in the absence of agonist (Figure 3). Similarly 1nM E2 and E1 were more sensitive to repression by raloxifene (Figure 4b) than were 1 nM (inactive levels) DES or E3, and the presence of E3 or DES reduced the ability of raloxifene to inhibit reporter activity by an order of 1,000 (compared to raloxifene properties in the absence of agonist – Figure 3). ICI 182,780 inhibited all the oestrogens equally (Figure 4c). However the presence of the non-stimulatory concentrations of oestrogens ICI 182,780 reduced the suppression of basal reporter activity (compare Figure 3 and Figure 4c) 100 fold. Taken together this indicates that despite inactivity of these agonists they nevertheless compete for binding to ER.

All the oestrogens tested were more sensitive to inhibition by raloxifene and ICI 182,780 than tamoxifen or trilostane. 10 μM trilostane (Figure 4d) inhibited reporter activity in the presence of all the oestrogens tested. However trilostane is stimulatory in the absence of oestradiol (Figure 3), and does not appear to compete with any of the other oestrogens tested, inhibition due to trilostane must occur via a different mode of action to tamoxifen, raloxifene and ICI 182,780.

### 3.6. Trilostane reduced the partial agonist properties of tamoxifen in MCF-7 cells

**Figure 5:**
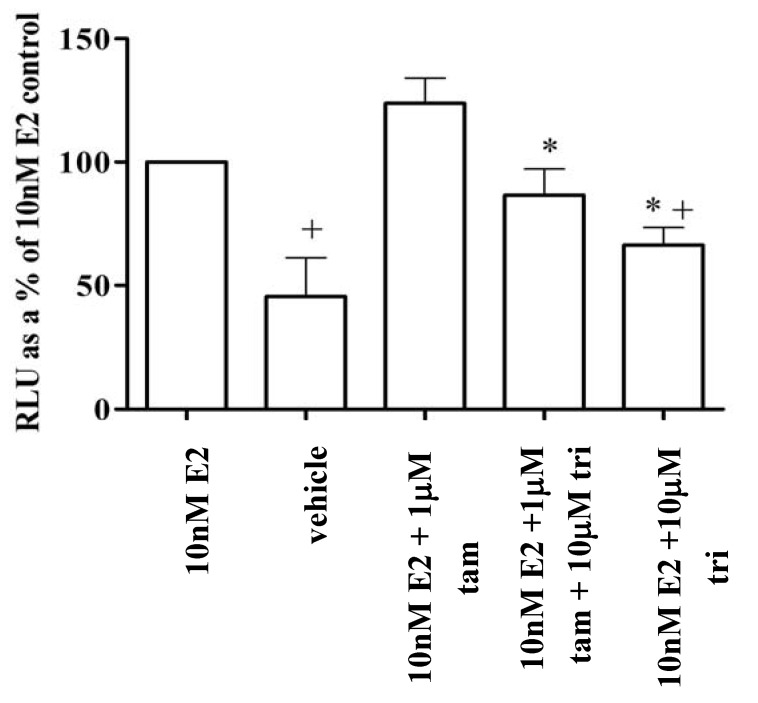
Trilostane inhibits oestrogenic activity of cells already stimulated with E2 and tamoxifen. Reporter activity was measured in the presence of combinations of stimulatory 10 nM E2, 10μM trilostane, with agonist/antiagonist properties, and 1μM tamoxifen, which is slightly inhibitory, and was expressed as a percentage of 10nM E2 control in MCF-7 cells. + indicates significant difference (P < 0.05) to 10nM E2 while * indicates significant repression (P < 0.05) compared to stimulation with E2 plus tamoxifen.

The activity and interaction between competitive and non-competitive antioestrogens was examined by the addition of a concentration of tamoxifen (1 μM), which is not significantly able to antagonise the peak activity of (10 nM) E2, to (10 μM) of trilostane. This concentration of trilostane is an agonist in the absence of other ligands but strongly inhibits 10 nM E2 (Figure 3 and Figure 4). It can be seen that 10 μM trilostane increased the antagonistic activity of 1 μM tamoxifen on 10 nM E2 (Figure 5), however trilostane had no additional effect in the presence of an antagonistic concentration (10 μM) of tamoxifen (data not shown). 

### 3.7. Oestrogenic activity is related to ERα, not ERβ

Clearly MCF-7 and T47D cells express ERα similarly (Figure 1), and, with some differences of detail, they respond similarly to stimulation (Figure 2). The two prostate cell lines, LNCaP and PNT1A neither contain ERα, nor do they respond to oestrogen. Though there is a good deal of variation in receptor and co-regulator content in the different cell types used (Figure 1), but only ERα appears to correlate with responsiveness. In particular, ERβ does not, since it is massively expressed in T47D cells compared with the low (though detectable) expression in the other cell types. Similarly, the high expression of PR in T47D cells, or of SRC-3 in MCF-7 cells confers no overall special modulation of the oestrogenic response.

Nevertheless, there are unexplained features of the responses. The relative binding affinities for oestrogens have been reported as a percentage of E2 (100%). In humans, E1 was 2% for ERα and 0.2% for ERβ, E3 was 29% (ERα), 80% (ERβ) and DES was 236% (ERα) and 221% (ERβ) [43]. In agreement with [42], the present results show that ligand-binding affinity does not necessarily predict gene transactivation and that reporter activity is regulated in a cell-specific manner. 

Candidate explanations for the differences in cell line specificity may be determined by examining the cellular content of ER and coregulator mRNA. Only two of the coregulators tested, both markers for disease progression, showed variation between the breast cancer cell lines (Figure 1). SRC-3 (AIB1), known to be upregulated in breast cancer and to correlate with tumour size [44], and which enhances ERα but not ERβ activity [45] was, as expected [46], highest in MCF-7 cells. REA, which also correlates with a worse breast cancer prognosis [47], was down regulated in T47D cells. Receptor, ER and PR, levels correspond to prognosis in breast cancer with a better prognosis for ER positive PR positive tumours [22]. The four cell lines tested contained various amounts of ERα, ERβ and PR mRNA (Figure 1). However, although ERβ and PR levels are generally inversely correlated [48], in this case, T47D cells, which had the most PR, also had the most ERβ. The two breast cancer lines, MCF-7 and T47D, (which both contained ERα, ERβ, and PR) responded to oestrogens (Figure 2), but the prostate lines, which contained only ERβ, did not. Yet even so the dose response curves for MCF-7 and T47D were different, with MCF-7 cells being more sensitive to stimulation by E2 than T47D (Figure 2), which, because ER preferentially heterodimerises [49] and is regulated by PR [50] is possibly due to the higher ERβ and PR content (Figure 1) of T47D cells.

Alternatively, since levels of ERα are the same in both MCF-7 and T47D cells, it is possible then that the high levels of AIB1 (SRC3) in MCF-7 cells (Figure 1d) contribute to their greater sensitivity to E2. There also may be some competition between high levels of REA (Figure 1d) with SRC-1 [51] in T47D cells resulting in their lower sensitivity. Thus the results here show certain aspects of the cell environment may be the explanation of the amelioration of reporter response in MCF-7 cells.

The cellular difference in reporter stimulation with oestrogens may also be due to splice variant [52,53,54] and mutated [55] ER present in MCF-7 cells. Similarly ER from T47D cells is a mixed population containing mutations [56] and splice variants [52,57]. In fact, because ER preferentially heterodimerise [58], inactive or partial ER may also be considered to be coregulators.

In addition it may be supposed that because trilostane is a inhibitor of 3β-hydroxysteroid dehydrogenase it may be acting via the glucocorticoid receptor to affect ER activity at the ERE. However, whilst other steroids do stimulate an ERE response in this system, we have preliminary data for aldosterone, 5αDHT, testosterone and cortisone (not shown) and they have different response curves in the different cell types. GR activity is not affected by trilostane [59]. Consequently it is unlikely that binding of trilostane to GR is directly affecting the ER response. 

**Figure 6:**
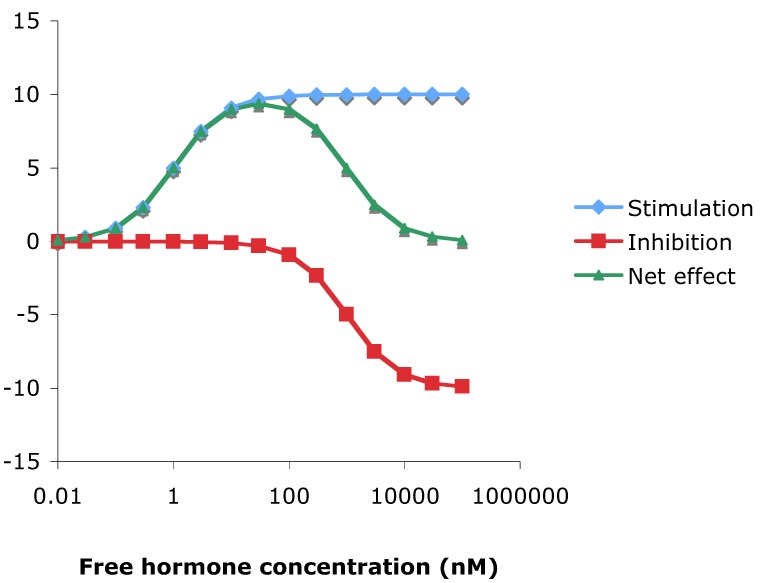
Predicted responses for a response to stimulation by a ligand that binds to a receptor with an activating site with Kd of 1nM, and to an inhibitory site with Kd of 1μM, assuming a receptor concentration of 10 nM.

Oestrogen stimulation of reporter activity was biphasic in both MCF-7 and T47D cells (Figure 2). Biphasic stimulation of MCF-7 cell proliferation has been previously observed for both E2 and DES [39] who suggested that it was due to toxicity at high concentrations. However the present results cannot be explained by toxicity as there was no concurrent reduction of the constitutively expressed *Renilla* luciferase. Though other mechanisms have been suggested [40,41], the existence of an alternative, inhibitory, ligand binding site more readily accounts for biphasic dose response curves, or hormesis, and this is illustrated in Figure 6. 

It has been reported that E2 is more active than E1, which is more active than E3 in promoting breast cancer cell line growth [60]. In the present experiment this pattern was reflected in reporter activity in MCF-7 cells. T47D cells, however, were more sensitive to E1 and E3 than to E2 or DES (Figure 2). It is also known that E1 is more efficient than E2 or E3 at recruiting SRC-1 to ERβ [61] and since ERβ is the major receptor in T47D cells this confirms a role for ERβ in oestrogenic signalling in this cell line.

Despite containing some ERβ neither prostate line was stimulated by any of the oestrogens tested (Figure 2). The low activity correlates to the absence of ERα (Figure 1), probably further inhibited by the low levels of AIB1 (Figure 1) in these cells. Repression of reporter activity in PNT1A may also be due to high levels of REA (Figure 1). ERβ from prostate is present as splice variants, such as the dominant negative ERβcx [62], which, like ER variants in MCF-7 and T47D cells, may influence reporter activity. A further possible factor in these low levels of reporter expression in these cells is the inability of ERβ to synergise on a multiple ERE [63]. In this case activation of T47D would still occur either because of the very high levels of ERβ or by heterodimerisation with ERα [49]. 

### 3.8. Novel activity of the antioestrogen trilostane

Trilostane is a non-competitive inhibitor [27] whereas tamoxifen, raloxifene and ICI 182,780 all compete for the ER ligand-binding pocket [22]. In the absence of E2, tamoxifen, raloxifene, and ICI 182,780 do not stimulate reporter activity (Figure 3), in fact they produce a non-permissive structural change [4,6,64] reflected in the repression of reporter activity below basal (Figure 3). There are reports of agonist activity of tamoxifen in the absence of oestrogen via AF-1 of ERα however this is cell type and promoter dependent [65] and depends on the level of differentiation of the cell [66]. In contrast, in these experiments, trilostane dose-dependently stimulated ER activity (Figure 3) so that, although it does not compete for E2 binding, it is able to bind to ER in the absence of oestrogens.

Trilostane was not only able to inhibit E2 and E3-stimulated transactivation but was able to repress reporter activity in cells treated with DES or E1 (Figure 4). This (along with the reduction in inhibition of reporter activity by tamoxifen, raloxifene or ICI 182,780 in the presence of these two oestrogens) demonstrates that, despite low activity of E1 or DES (Figure 3), these agonists are binding to ER in MCF-7 cells. While the activity of tamoxifen, raloxifene and ICI 182,780 is reduced by competition with the same weak ER agonists (compare Figure 3 and Figure 4), the antagonist effect of trilostane does not rely on competition with any of the oestrogens used, suggesting that it acts via a second, regulatory, binding site. 

Trilostane was also able to repress the agonist activity of tamoxifen and E2 together (Figure 5) demonstrating that trilostane does not compete with tamoxifen either. Trilostane may therefore be useful, not only in treating ER positive tumours, but also in the treatment of tamoxifen-stimulated tumours, or a use in combinatorial therapy.

Ligand binding changes the coactivator- and coregulator-binding surface [67] therefore the availability of ligand at the binding-pocket and cell environment interact to produce the oestrogenic capacity of a ligand. A second-binding site could regulate the ligand-binding domain, either by causing a conformational change to physically interfere with ligand binding, or by altering co-regulator association with the receptor. 

### 3.9. Model of a regulatory allosteric ligand binding site on ER

Throughout the literature there is a great deal of evidence for a second, allosteric, ligand-binding site. In yeast oestrogen reporter systems, xenoestrogens were 100 times more potent in combination than alone and, although unable to stimulate the reporter system or to antagonise oestradiol stimulation, chlordane was able to enhance antioestrogen activity [68]. Also in yeast E2 and E1 were non-competitively synergistic and E2 enhanced binding of E1 [28]. 

A second binding site can also be suggested for the action of phytoestrogens, which do not compete for the hormone-binding pocket [69,70] and for other xenoestrogens such as polynuclear aromatic hydrocarbons, which non-competitively inhibit E2 stimulation of a reporter system in yeast [71]. In addition, in human endometrium cancer cells, tetrahydroxychrysene derivatives stimulated gene transcription via ERα but were increasingly antagonistic to ERβ with increasing size of side groups [33,32] indicating that there was a specific binding site. 

Further evidence for a second binding site includes Jensen *et al.* [29] who realised that ER bound twice as much tamoxifen as E2 and proposed a model of second site binding where antioestrogens bound to a second negative binding site unavailable to E2. A second site model has also been suggested for the mineralocorticoid receptor [72].

### 3.10. Theoretical consideration of hormesis

To simulate the effect of a second, inhibitory, site that binds ligand at a considerably lower affinity than the main activating ligand binding domain, use was made of the formula:

                                          [HR] = [Hf] X [Rf]/([Hf] + Kd),

where HR = hormone receptor complex, Hf = free hormone, Rf = unoccupied receptor, and Kd = dissociation constant. Over a range of hormone and receptor concentrations, this may be predicted to be directly related to hormone efficacy [73]. This gives predictions of stimulatory and inhibitory monotonic responses, which when combined can deliver pseudo dose-response curves remarkably similar to those found in practice, compare Figure 2 and Figure 6. 

## 4. Conclusions

In total then, the results presented here may be explained by the presence of a second binding site because:

The dose response curves to oestrogens were biphasic such that stimulation of the reporter became auto-inhibitory at high concentrations (Figure 2), consistent with theoretical considerations (Figure 6) 

High concentrations of oestrogens were able to inhibit reporter activity in cell lines which were unable to positively respond to oestrogens (Figure 2)

Trilostane was an agonist in the absence of oestrogen (Figure 3) but was non-competitively antioestrogenic (Figure 4) 

Trilostane was able to repress reporter activity in the presence of non-inhibitory concentrations of tamoxifen but not when tamoxifen itself was inhibitory (Figure 5)

Therefore, one reason that stimulation of the oestrogen receptor by agonists invariably reached a maximum, then declined (Figure 2), could be because of repression via an allosteric site, and, in contrast to the competitive repression exerted by antioestrogens such as tamoxifen, raloxifene and ICI 182,780 (Figure 4), in this model, the antagonism of trilostane occurs via the second site.

There is therefore a huge amount of control available to ensure that the right oestrogen responsive gene is switched on, in the right tissue, at the right time. A better understanding of this process would hopefully lead to an ability to restore cell cycle control in hormone sensitive tumours. 
